# Improved Diabetic Foot Ulcer Outcomes in Medicaid Beneficiaries with Podiatric Care Access

**DOI:** 10.3390/diabetology5050036

**Published:** 2024-10-10

**Authors:** Ivan Y. Luu, Alexander T. Hong, Ashton Lee, Juan C. Arias, Chia-Ding Shih, David G. Armstrong, Tze-Woei Tan

**Affiliations:** 1 Keck School of Medicine, University of Southern California, Los Angeles, CA 90033, USA; 2 San Antonio Vein and Artery Surgery, San Antonio, TX 78207, USA; 3 College of Medicine, University of Arizona, Tucson, AZ 85724, USA

**Keywords:** diabetic foot ulcer, amputation, foot infection, podiatry, Medicaid

## Abstract

**Objectives:**

This study aims to examine the association between state Medicaid coverage of podiatry services and the outcomes of beneficiaries with new diabetic foot ulcers (DFUs).

**Methods:**

Medicaid beneficiaries who developed a DFU between 2010 and 2015 were identified using the PearlDiver claims database. The states were categorized into covered states (CS) and non-covered states (NCS) based on podiatric coverage during the study period. The outcomes included major amputation, minor amputation, and hospitalization due to foot infection within 12 months of index diagnosis. Logistic regression was used to assess the association of state coverage type and outcomes, controlling for age, sex, and the Charlson Comorbidity Index (CCI).

**Results:**

Our study included 16,905 Medicaid beneficiaries who developed new DFUs: 14,748 in CS and 2157 in NCS. The overall major amputation rate was 2.6%. The risk of major amputation was 48% lower among Medicaid beneficiaries in CS (OR 0.52, 95% CI 0.31–0.90) than in NCS. The Medicaid beneficiaries in CS had a 24% lower risk of hospitalization for foot infection (OR 0.76, 95% CI 0.67–0.85) but had a 58% higher risk of minor amputation (OR 1.58, 95% CI 1.22–2.07) than in NCS.

**Conclusions:**

Medicaid coverage of podiatry services might be associated with lower rates of major amputation and reduced risk of hospitalization for foot infection.

## 1. Introduction

According to the Center for Disease Control, 38.1 million adults aged 18 years or older, or 14.7% of all U.S. adults, had diabetes [1]. Among those U.S. adults who met the laboratory criteria for diabetes, 22.8% of them were not aware or did not report having diabetes. The disease is the eighth leading cause of death in the U.S., and in 2022, it was responsible for 101,209 deaths [1].

Diabetic foot ulcers (DFUs) are common among the diabetic population, are associated with high mortality and morbidity rates, and are extremely costly. DFUs are wounds that penetrate through the dermis and are located below the ankle in diabetic patients [2,3]. It is estimated that up to 34% of people with diabetes will develop DFUs [4,5]. For patients with DFUs, the annual risk of lower extremity amputation (LEA) ranges from 0.25 to 1.8%, and even after the ulcer heals, there is a 12% risk of LEA within five years [4]. Patients with DFUs have a five-year mortality of 40%, which increases to approximately 60% in those who undergo amputation [6]. The rate of hospital admissions for DFUs is rising, and DFUs and their complications account for the majority of major and minor amputations performed on an inpatient basis [4,7,8]. The direct costs of treating DFUs in the US are estimated to be greater than USD 9 billion annually, with additional costs associated with treating diabetes [9-11].

Fortunately, studies have shown that prevention of DFUs and their related amputations is possible and cost-saving. Preventive foot care and timely treatment of DFUs can prevent adverse outcomes in people with diabetes [12-15]. Guidelines from the American Diabetes Association and the joint recommendation from the American Podiatric Medical Association and Society for Vascular Surgery recommend at least one annual foot examination by trained healthcare providers for all patients with diabetes, depending on their risk of developing DFUs [16,17]. An interdisciplinary approach to managing and preventing DFUs has proven to be paramount [18-20]. Podiatrists play a fundamental role in the collaborative treatment of DFUs, focusing on wound management and patient education, particularly regarding foot self-care habits before and after the diagnosis of DFUs [21]. Services integrating podiatric medicine and surgery as part of the outpatient multidisciplinary DFU care have resulted in reduced major amputation rates [22,23]. Additionally, outpatient pre-DFU foot and ankle care by podiatrists prior to the diagnosis of DFU has been associated with lower risks of major amputation and hospitalization for foot infections [24].

The development of DFUs is closely related to the social determinants of health and one’s socioeconomic status. Individuals who receive Medicaid, a federal and state-supported safety net program providing healthcare coverage to low-income Americans, are at high risk for complications once they develop a DFU [25-27]. Studies have shown that, compared to those with Medicare or commercial insurance, Medicaid beneficiaries with DFUs have higher rates of LEA and incur higher care costs [28]. Despite this, Medicaid coverage for podiatric care varies by state, with some states eliminating podiatric services reimbursement as a cost-cutting measure [29]. While the discrepancy in podiatric preventive care exists, DFU-related LEA rates and hospitalizations due to infection are only inferred based on the epidemiological analyses [30].

The purpose of this study was to analyze the differences in LEA rates and hospitalizations for foot infection between Medicaid beneficiaries in states that include podiatric services in their coverage and those in states that do not.

## 2. Materials and Methods

### 2.1. Data Source

We retrospectively queried the PearlDiver database (PearlDiver Technologies, Inc., Fort Wayne, IN, USA), a fee-based dataset that has been widely used in health economics research from 2010 to 2018. As of November 2021, the database contained 122 million distinct patients from all 50 states and territories in the United States from 2010 to 2018. Payer types included commercial, Medicare, Medicaid, and cash. The analysis for this study was performed using the built-in statistical analysis software. Our study was approved by the Institutional Review Board of the University of Arizona (ID 00000155), and the requirements for informed consent requirements were waived due to the use of de-identified data. This study followed the Strengthening the Reporting of Observational Studies in Epidemiology (STROBE) reporting guidelines.

### 2.2. Patient Population

The participants who were Medicaid beneficiaries with a new incidence of DFU from 2010 to 2015 were identified using the International Classification of Diseases, Ninth Revision (ICD-9) codes. The inclusion criteria for the study required that the patients were adults aged 20–69 years with a newly diagnosed DFU (no previous diagnosis of DFU within 12 months of the index diagnosis), were Medicaid beneficiaries, and had continuous enrollment in the database for at least 12 months prior to the index diagnosis and 36 months later. Patients with a history of previous major amputation on either leg were excluded from the study because one of the major outcomes we studied was major amputation.

### 2.3. Data Points Collected

The following comorbidities were identified in the Mariner database: age, Charlson Comorbidity Index (CCI), chronic obstructive pulmonary disease (COPD), cerebrovascular disease (CVD), chronic kidney disease (CKD), chronic heart failure (CHF), coronary artery disease (CAD), hypertension (HTN), obesity, depression, renal failure, obesity, severe liver disease, peripheral artery disease (PAD), gangrene, and infection.

### 2.4. Outcome Measures

The outcomes examined were major amputation, minor amputation, and hospital admission within 12 months of the index diagnosis of diabetic foot infection. Patient outcomes were stratified based on whether they lived in states with podiatric services coverage for their Medicaid programs (CS) or were without such coverage in non-covered states (NCS) during the study period. The NCS group included Medicaid beneficiaries from five states: AL, AZ, NV, NY, and WY. The CS group included Medicaid beneficiaries in 40 states: AK, NM, TX, UT, CO, DE, DC, FL, GA, ID, IL, IA, KS, KY, LA, ME, MD, MA, MI, MN, MS, MO, MT, NE, NH, NJ, NC, ND, OH, OK, OA, RI, SD, RN, VT, VA, WA, WV, and WI. The states in which Medicaid policies on the coverage of podiatric care changed during the study period were not included in the analysis. Coverage policies were identified using the Kaiser Family Foundation (KFF) Medicaid benefits database [31].

### 2.5. Statistical Analysis

Data were reported as the mean for continuous descriptive variables, median for ordinal descriptive variables, and proportion for categorical variables. We used the chi-squared test to identify differences in the baseline demographics and characteristics for the categorical variables. Multivariable logistic regression analysis was performed to study the association between Medicaid podiatric services coverage and outcomes, including major amputation, minor amputation, and hospitalizations within 12 months of index diagnosis for foot infection, controlling for age, gender, CCI, and medical comorbidities. Associations were presented as adjusted odds ratios (ORs) with corresponding 95% confidence intervals (CIs). A *p*-value of less than 0.05 was considered significant.

## 3. Results

A total of 16,905 Medicaid beneficiaries with a new DFU were included in the study cohort and divided into two groups based on state-level Medicaid coverage of podiatric services: CS (*n* = 14,748) and NCS (*n* = 2157). Although there was no difference in mean age between the groups, the CS group had a higher percentage of patients aged 40–54 (44.0% vs. 24.1%), while the NCS group had more patients in the 55–65 age group (55.5% vs. 35.9%) (both *p* < 0.001) (Table 1). The patients in the NCS group had higher CCI scores (2.6 vs. 2.3, *p* < 0.01) and were more likely to have CVD (32.8% vs. 30.6%, *p* = 0.04) or severe liver disease (5.2% vs. 4.5%, *p* < 0.01). The patients in the CS group were more likely to have CKD (35.9% vs. 30.0%, *p* < 0.01) and renal failure (24.5% vs. 21.6%, *p* < 0.01). While there was no significant difference in PAD and gangrene, the patients in the NCS group were more likely to have a foot infection during their index DFU diagnosis (75.9% vs. 71.5%, *p* < 0.01).

### 3.1. Logistic Regression for Major Amputation

**Figure 1.**
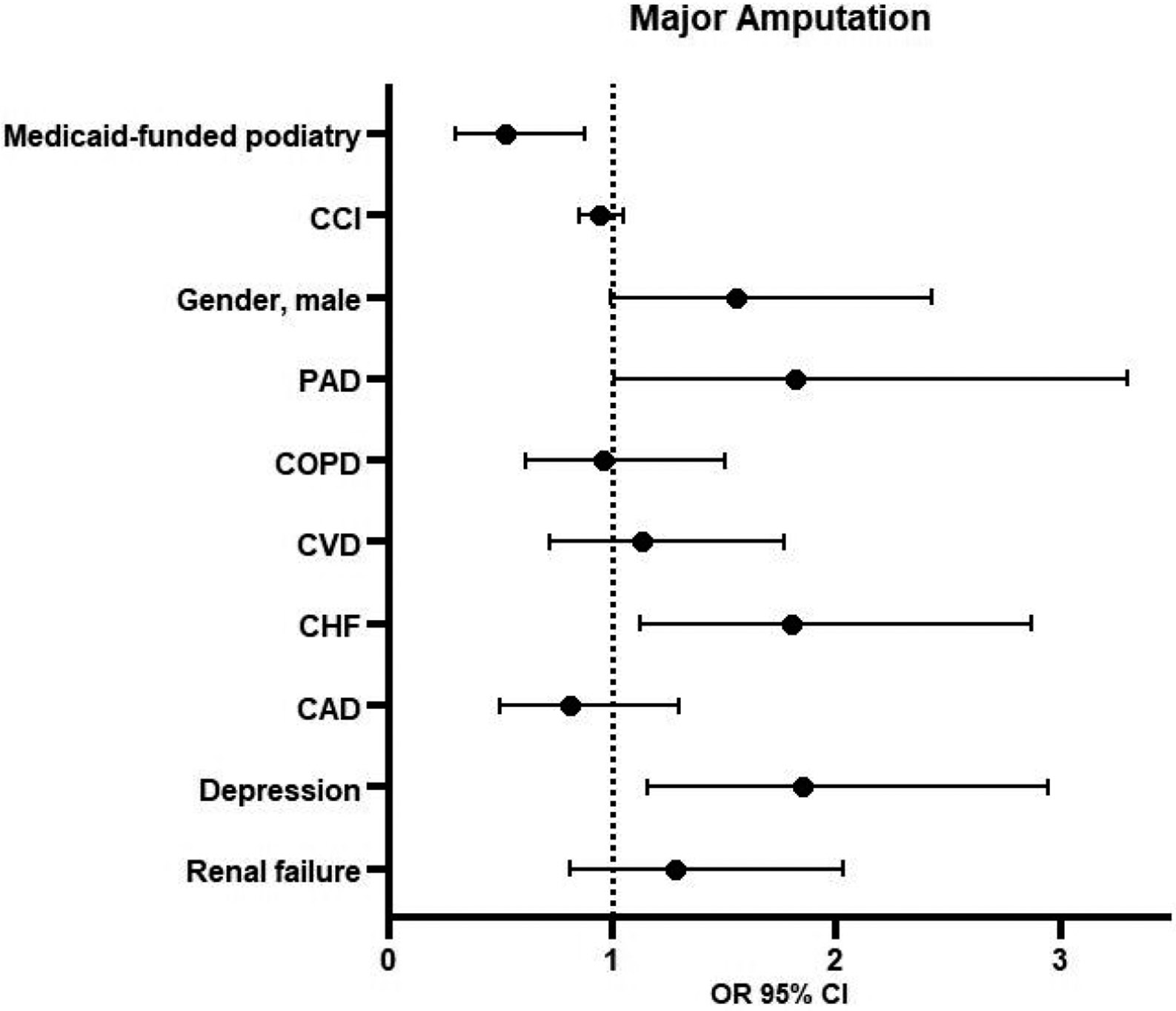
Logistic regression for major amputation. Abbreviations: CCI = Charlson Comorbidity Index, PAD = peripheral artery disease, COPD = chronic obstructive pulmonary disease, CVD = cerebrovascular disease, CHF = chronic heart failure, CAD = coronary artery disease, OR = odds ratio, CI = confidence interval.

**Figure 2.**
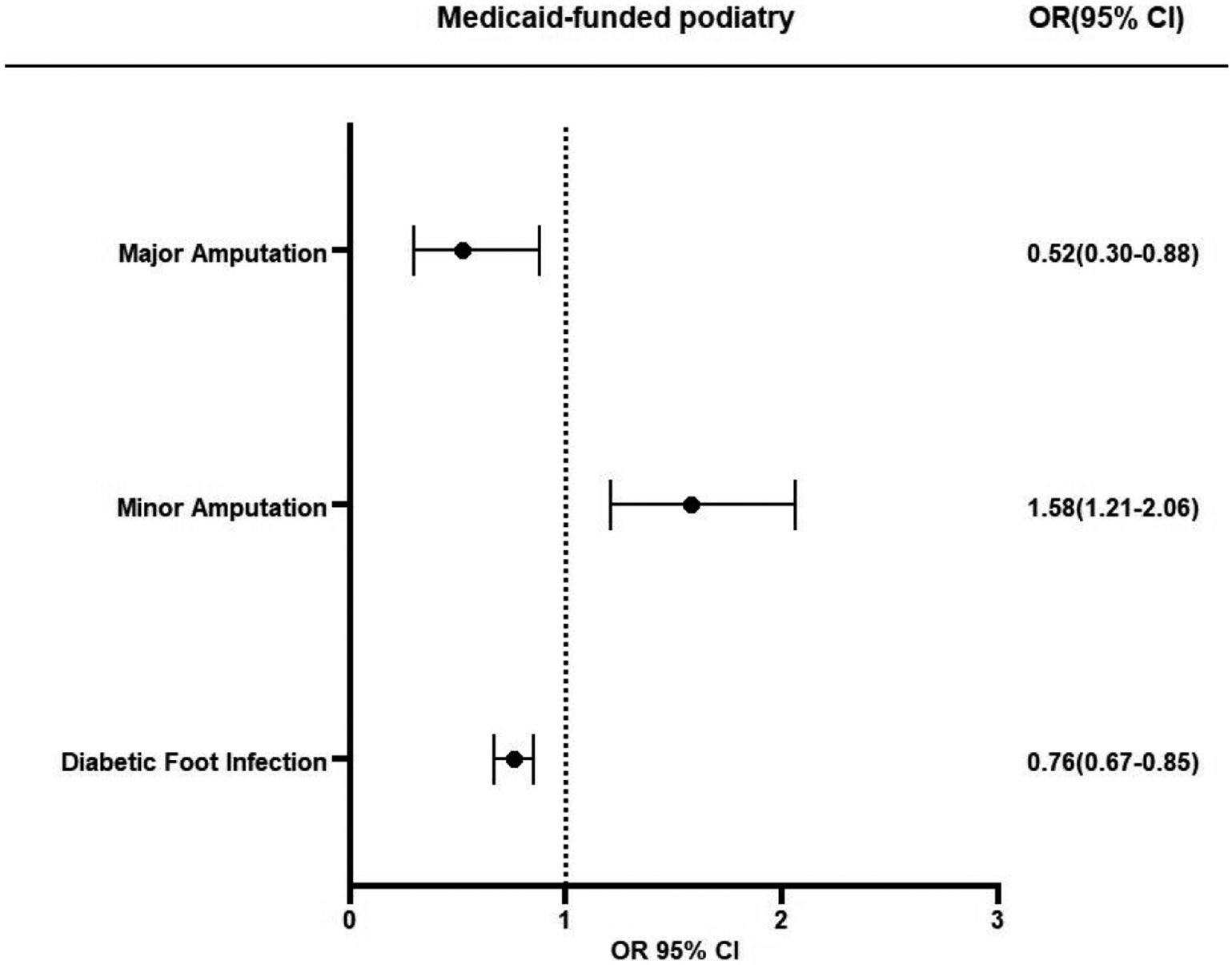
Association of podiatric services coverage for their Medicaid programs and major amputation, minor amputation, and hospitalizations for foot infection after DFU diagnosis. Abbreviations: OR = odds ratio, CI = confidence interval.

Multivariable logistic regression demonstrated that the Medicaid beneficiaries in the CS group were 48% less likely to undergo major amputation (OR 0.52, 95% CI 0.3–0.88, *p* = 0.01) than the NCS group (Figures 1 and 2). Other factors associated with major amputations included CHF (OR 1.80, 95% CI 1.12–2.87, *p* = 0.01), depression (OR 1.85, CI 1.16–2.95, *p* = 0.01), and PAD (OR 1.82, 95% CI 1.01–3.30, *p* = 0.05).

### 3.2. Logistic Regression for Minor Amputation

**Figure 3.**
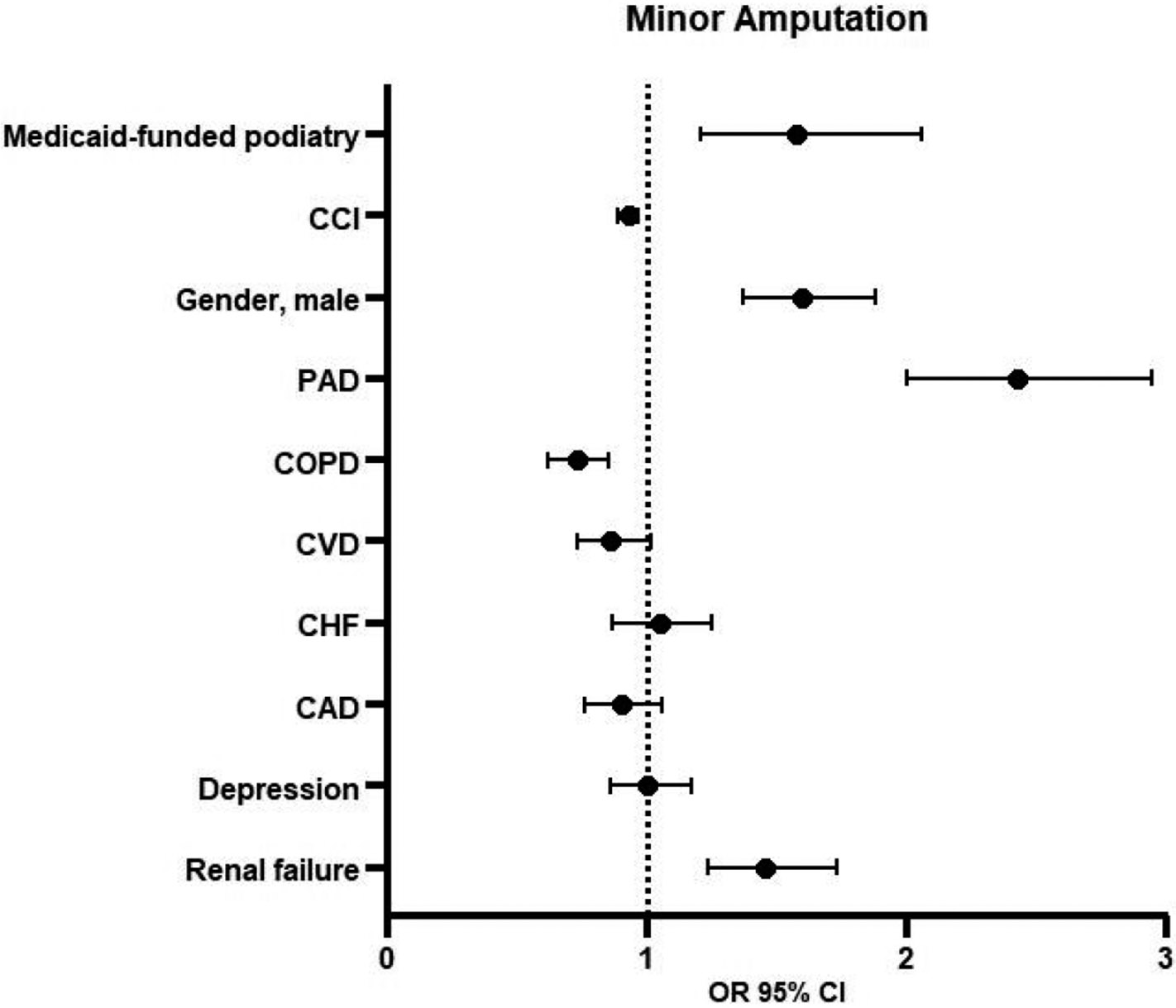
Logistic regression for minor amputation. Abbreviations: CCI = Charlson Comorbidity Index, PAD = peripheral artery disease, COPD = chronic obstructive pulmonary disease, CVD = cerebrovascular disease, CHF = chronic heart failure, CAD = coronary artery disease, OR = odds ratio, CI = confidence interval.

Logistic regression showed that the Medicaid beneficiaries in the CS group were 58% more likely to undergo minor amputation (OR 1.58, 95% CI 1.21–2.06, *p* = 0.01) (Figures 2 and 3). Other factors associated with minor amputations were CCI (OR 0.93, 95% CI 0.89–0.97, *p* = 0.01), male gender (OR 1.60, 95% CI 1.37–1.88, *p* = 0.01), PAD (OR 2.43, 95% CI 2.00–2.95, *p* = 0.01), COPD (OR 0.73, 95% CI 0.62–0.85, *p* = 0.01), and renal failure (OR 1.46, 95% CI 1.24–1.73, *p* = 0.01). The patients with COPD had 27% lower risk of minor amputation (OR 0.73, 95% CI 0.62–0.85, *p* < 0.01).

### 3.3. Logistic Regression for Hospitalization Due to Foot Infection within 12 Months of DFU Diagnosis

**Figure 4.**
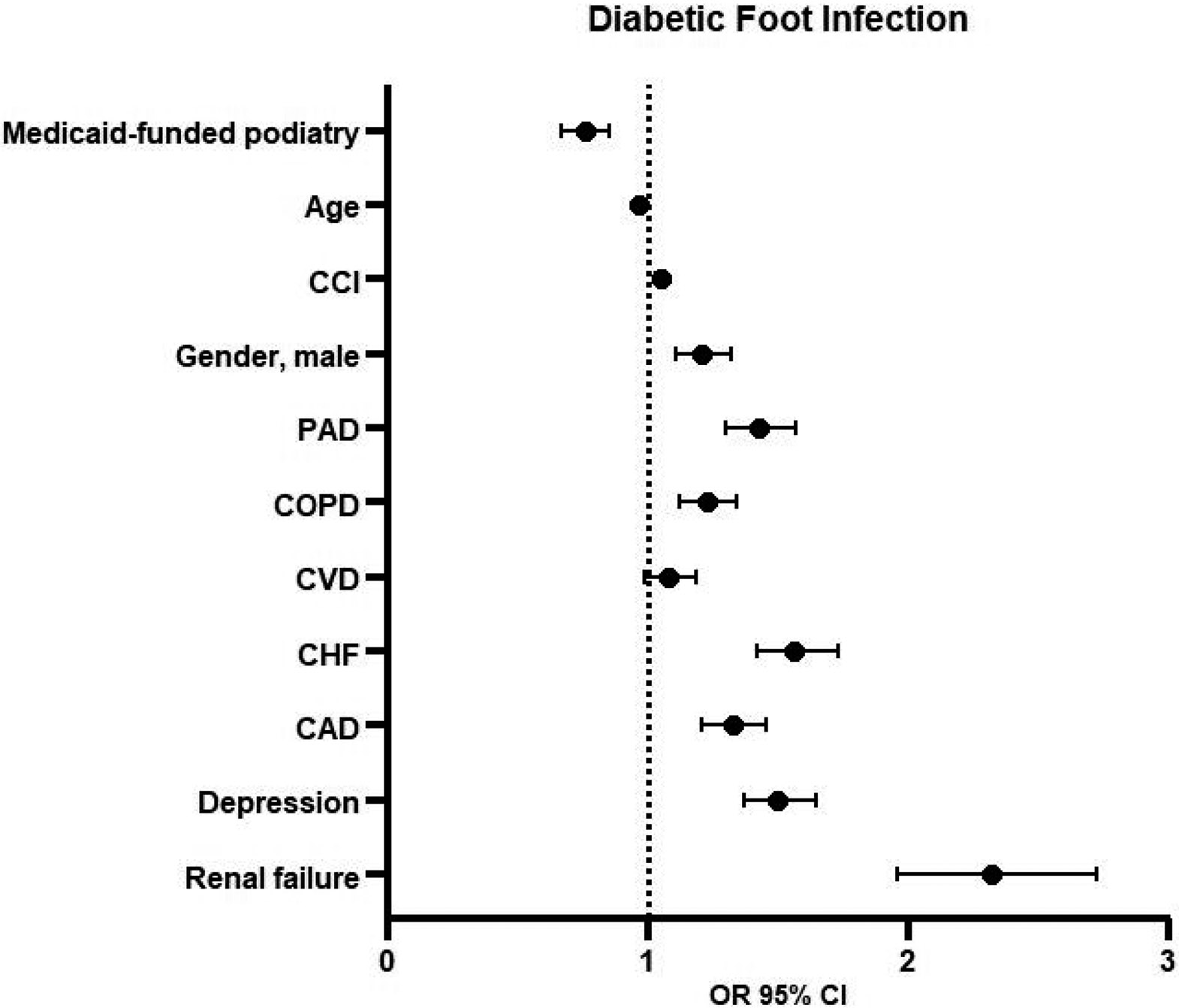
Logistic regression for hospitalizations for foot infection within 12 months after index diabetic foot ulcer (DFU) diagnosis. Abbreviations: CCI = Charlson Comorbidity Index, PAD = peripheral artery disease, COPD = chronic obstructive pulmonary disease, CVD = cerebrovascular disease, CHF = chronic heart failure, CAD = coronary artery disease, OR = odds ratio, CI = confidence interval.

The Medicaid beneficiaries in the CS group were 24% less likely to be hospitalized within 12 months for foot infection (OR 0.76, 95% CI 0.67–0.85, *p* = 0.01) (Figures 2 and 4). Other factors associated with diabetic foot infection were age (OR 0.97, 95% CI 0.97–0.98, *p* < 0.01), CCI scores (OR 1.05, 95% CI 1.03–1.07, *p* < 0.01), male gender (OR 1.21, 95% CI 1.11–1.32, *p* < 0.01), PAD (OR 1.43, 95% CI 1.30–1.57, *p* < 0.01), COPD (OR 1.23, 95% CI 1.12–1.34, *p* < 0.01), CHF (OR 1.56, 95% CI 1.42–1.73, *p* = 0.01), CAD (OR 1.33, 95% CI 1.21–1.46, *p* < 0.01), depression (OR 1.50, 95% CI 1.37–1.65, *p* < 0.01), and renal failure (OR 2.32, 95% CI 1.96–2.73, *p* < 0.01).

## 4. Discussion

Our study analyzed the outcomes of Medicaid beneficiaries with newly diagnosed DFUs, comparing states with and without podiatric services in their Medicaid programs. Those living in states with podiatric coverage had a lower risk of major amputations but a higher risk of minor amputations, suggesting that access to podiatric care might enable foot care that prevents severe limb complications such as major amputations and hospitalizations for foot infection. These findings underscore the benefits of including standardized podiatric care in Medicaid programs nationwide to improve outcomes for the vulnerable populations that are at risk of DFUs and related LEAs.

National guidelines recommend that patients with diabetes undergo at least an annual foot examination by a trained medical professional to evaluate the risk of DFUs and amputation [16]. Numerous strategies to reduce amputation have been proposed for patients with diabetic foot disease, including patient education [32], offloading to promote DFU healing and prevent recurrence after healing [33], and foot and ankle care by a podiatrist [34]. Prior studies have suggested that only 30% of patients receive preventative diabetic foot care before developing ulceration [35]. Evidence also indicates that multidisciplinary care, including podiatric services, reduces amputations after DFU development [36]. Structured diabetic foot services which include screening and podiatric care positively impact the rates of major amputation [37,38].

Podiatry plays a critical role in the prevention and management of diabetic foot complications. As of September 2020, 42 states included podiatric coverage for Medicaid beneficiaries and 5 states did not [39]. Among the 42 states that provided podiatric services, 25 of these states required prior authorization, 19 had out-of-pocket costs when accessing podiatric services, and 26 states imposed limits on the number of visits [39]. The elimination of podiatry reimbursement in Arizona in 2010 led to a significant increase in hospitalizations for DFUs and associated cost, with every USD 1 saved in reimbursement resulting in a USD 48 increase in hospitalization costs [40].

Maintaining Medicaid access is crucial to provide healthcare coverage among vulnerable populations, including low-income families, qualified pregnant women and children, and individuals receiving Supplemental Security Income (SSI) [41-44]. Expansion of Medicaid eligibility under the Affordable Care Act (ACA) in 2014 has been associated with reduced disparity in surgical access, greater care access, and improved health status in states with high diabetes prevalence [45]. For individuals with diabetes, the expansion has led to increased routine foot care [46]. However, no study that we are aware of has evaluated the direct effects of Medicaid coverage of podiatric services and DFU outcomes.

In this retrospective cohort study, we demonstrate that Medicaid access to podiatric services reduces major amputation and hospitalizations for foot infection following DFU diagnosis. To our knowledge, this is the first study to show the protective benefit of Medicaid-covered podiatric services in patients with DFUs. After controlling for medical comorbidities, such as renal failure and PAD, our multivariate analysis revealed that Medicaid beneficiaries in the CS group, compared to the NCS group, had a 48% lower risk of major amputation and were 24% less likely to be hospitalized within 12 months of foot infection. Interestingly, the risk of minor amputation was 58% higher in the CS group. This increased rate of minor amputations could be attributed to early and aggressive surgical management, which helps prevent progression to major amputations.

This study has several limitations. First, it is a retrospective review based on claims data. Second, we did not verify whether the participants received podiatric care, relying instead on the differences in state-funded podiatry services through Medicaid. Nonetheless, our previous study using the same dataset showed that patients who received outpatient pre-ulcerative podiatrist foot care within 12 months of a new DFU had lower risks of major amputation [47]. Third, although we performed multivariable analyses to control for potential confounding factors, such as medical comorbidities, social determinants that may affect access to foot care, such as transportation and language barriers, were not accounted for. Future analyses will explore how specific Medicaid policies on podiatric services impact pre- and post-ulcer diabetic foot care, healthcare utilization such as ED visits, and long-term DFU outcomes.

## 5. Conclusions

Our study shows that Medicaid coverage for podiatry services is associated with improved outcomes for beneficiaries with newly diagnosed DFUs. Medicaid beneficiaries in states with podiatry coverage had lower rates of major amputations and fewer hospitalizations for foot infections. These findings underscore the critical role of podiatric care in managing patients at risk of DFUs and highlight the need for podiatry services to be considered essential coverage under Medicaid.

## Figures and Tables

**Table 1.** Baseline characteristics and comorbidities of Medicaid beneficiaries in states with podiatric services coverage for their Medicaid programs (CS) or without such coverage (NCS).

	Total	CS (*n* = 14,748)	NCS (*n* = 2157)	*p*-Value
Age (mean), years	48.9	48.9	49.0	0.70
Age group, *n* (%)				<0.001
<40	3360 (19.9)	2960 (20.1)	400 (18.5)	
40–54	6972 (41.2)	6453 (44.0)	519 (24.1)	
55–65	6502 (38.5)	5305 (35.9)	1197 (55.5)	
Male gender, *n* (%)	8556 (50.6)	7521 (51.1)	1035 (48.0)	0.03
CCI (mean)	2.3	2.3	2.6	<0.01
0–4	14,862 (87.9)	13,044 (88.4)	1818 (84.3)	
5–9	1755 (10.4)	1473 (10.0)	282 (13.1)	
10–14	226 (1.34)	207 (1.6)	19 (0.9)	
CVD, *n* (%)	5220 (30.9)	4513 (30.6)	707 (32.8)	0.04
CHF, *n* (%)	4126 (24.4)	3628 (24.6)	498 (23.1)	0.13
COPD, *n* (%)	9258 (64.8)	8084 (54.8)	1174 (54.4)	0.75
CAD, *n* (%)	6793 (40.2)	5973 (40.5)	820 (38.0)	0.03
Hypertension, *n* (%)	15,299 (90.5)	13,362 (90.6)	1937 (89.8)	0.27
CKD, *n* (%)	6142 (36.3)	5495 (35.9)	647 (30.0)	<0.01
Renal failure, *n* (%)	4079 (24.1)	3613 (24.5)	466 (21.6)	<0.01
Obesity, *n* (%)	9876 (58.4)	8642 (58.6)	1234 (57.2)	0.22
Severe liver disease, *n* (%)	776 (4.6)	664 (4.5)	112 (5.2)	<0.01
PAD, *n* (%)	7306 (43.2)	6342 (43.0)	964 (44.7)	0.15
Gangrene, *n* (%)	1999 (11.8)	1755 (11.9)	244 (11.3)	0.45
Infection, *n* (%)	12,182 (72.1)	10,545 (71.5)	1637 (75.9)	<0.01

Abbreviations: CCI = Charlson Comorbidity Index, CVD = cerebrovascular disease, CKD = chronic kidney disease, CHF = chronic heart failure, COPD = chronic obstructive pulmonary disease, CAD = coronary artery disease, PAD = peripheral artery disease.

## Data Availability

The raw data supporting the conclusions of this article will be made available by the authors on request.
